# Traditional Health Practitioners-Initiated HIV Testing and Counselling: Perspectives from Health Care Workers, Healers and Clients in Rural South Africa

**DOI:** 10.1007/s10461-025-04761-4

**Published:** 2025-05-31

**Authors:** Jennifer Hove, Praise Mnisi, Wonderful Mabuza, Ryan G. Wagner, Tshegofatso Seabi, Carolyn M. Audet

**Affiliations:** 1 Faculty of Health Sciences, SA MRC/Wits Rural Public Health and Health Transitions Research Unit (Agincourt), School of Public Health, University of Witwatersrand, 27 St Andrew Rd, Johannesburg, South Africa; 2 Vanderbilt Institute for Global Health, Vanderbilt University, 2525 West End Avenue, Suite 750, Nashville, TN 37203-1738 USA

**Keywords:** HIV testing, South Africa, Traditional Health Practitioners, Rural

## Abstract

A considerable proportion of people living with HIV remain unaware of their HIV status. In South Africa, individuals in rural areas often receive health care from both allopathic providers and traditional health practitioners (THPs). Our team piloted a task-shifting pilot study to determine the feasibility of THP-led HIV counselling and testing. Our team conducted semi-structured, qualitative interviews with healthcare workers, THPs who completed HIV counselling and testing training, and clients who received a positive test result. Interviews focused primarily on participant opinions about, and experiences with, the intervention and their perspectives on how (and if) this partnership should be maintained. Transcripts were analyzed by two authors, employing phenomenological thematic coding using the socio-ecological model. Our team conducted 33 interviews: 12 with healthcare workers, 11 with THPs, and 10 with clients who received a positive test result. A combination of deeply rooted cultural beliefs in the value of traditional medicine and dissatisfaction with the allopathic health services led most participants to value the introduction of THP-led testing. Concerns about THPs’ ability to deliver testing was raised by healthcare workers, but clients reported that THPs delivered testing with fidelity. Several participants spoke about the importance of integrating THPs into the national health system to ensure they had access to test kits and support from clinicians. Integrating THPs into the health system will require the organization of a government-led training program and the creation of an official position for trained THPs within their local clinic.

## Introduction

 In South Africa, approximately 7.8 million people were estimated to be living with HIV (PLHIV) in 2020 [1–3]. The results of the 2022 survey showed significant progress in moving toward achieving the Joint United Nations Programme on HIV/AIDS (UNAIDS) 95-95-95 targets [2]. Unfortunately, despite a significant increase in ART coverage over the preceding two decades and substantial strides in reducing HIV incidence and mortality rates, over a quarter of diagnosed PLHIV were not receiving antiretroviral therapy (ART) on a nationwide scale in 2020 [2, 4]. A considerable proportion of PLHIV remain unaware of their HIV status [1, 4, 5]. In 2022, 90% of South Africans living with HIV knew their status [2, 3]. Among them, 91% were receiving treatment, and 94% of those receiving treatment had achieved viral suppression [2, 4]. Nonetheless, data indicate persistent inequalities within South Africa’s HIV landscape [2–4, 6]. Individuals residing in marginalized rural communities are nearly twice as likely to be HIV-positive compared to their urban counterparts and are less likely to receive an HIV test [2, 4].

In South Africa, like many other African nations, approximately 80% of individuals residing in rural areas utilize a combination of allopathic medicines available at hospitals and clinics, along with traditional or alternative medicines offered by traditional health practitioners (THPs) [7–11]. THPs, commonly known locally as traditional healers, *sangomas*, or *inyangas*, are recognized and respected members of local communities [12, 13]. In South Africa, there are more than 200,000 THPs, primarily residing in rural areas [8]. THPs are a significant source of healthcare for many individuals [8, 9, 14]. Research indicates a preference among individuals to seek the services of THPs for various health conditions, including symptoms related to HIV/AIDS [11, 12, 15]. Numerous factors influence the utilization of THPs, including perceived accessibility, low cost, alignment with sociocultural, religious, and spiritual beliefs, as well as dissatisfaction with allopathic medicine [5, 8].

Recognizing the importance of the role of THPs, this paper reflects on the integration of THPs into HIV testing and counseling services in the Bushbuckridge Sub-district. HIV diagnosis serves as a crucial step in linking HIV-positive individuals to necessary care and treatment [5, 16]. Timely linkage to care and initiation of ART play a pivotal role in mitigating the risks associated with HIV-related morbidity, mortality, and transmission [5, 17]. However, the uptake of HIV testing remains suboptimal in rural communities, posing significant challenges [1, 5, 7]. Many individuals prefer consulting THPs over visiting health clinics due to structural barriers, including reluctance toward HIV testing [7, 8]. With their deep-rooted understanding of cultural beliefs and practices, and their role as custodians of indigenous knowledge, THPs are regarded as trusted figures in addressing various physical, mental, and spiritual ailments [7, 14, 18]. Acknowledging the importance of leveraging their influence, discussions surrounding the integration of THPs into HIV testing and counselling services have emerged to bridge gaps in healthcare access and promote culturally sensitive care delivery. This aligns with the recommendations of the World Health Organization (WHO), which advocates for augmenting conventional facility-based HIV testing with community-based HIV testing services to enhance testing coverage [19]. This paper explores the perspectives and experiences of healthcare workers, THPs, and THP clients who received a positive test result during the trial. Specifically, we aimed to assess the success of counseling and testing training, service delivery (including referral for those who tested HIV positive) and the ways in which the community and health system professionals reacted to the delivery of THP-led HIV testing.

## Methods

### Qualitative Data Collection

#### Setting, Participants and Sampling

The research was conducted in the Bushbuckridge Sub-district, within the Mpumalanga province of South Africa, approximately 500 km northeast of Johannesburg. Specifically, the study took place within the South African Medical Research Council (MRC)/University of the Witwatersrand (Wits) Rural Public Health and Health Transitions Research Unit in Agincourt, where a team of researchers has established a Health and Socio-Demographic Surveillance System (HDSS). Established in 1992, the HDSS conducts annual updates, collecting data on vital events such as births, deaths, migration, and socioeconomic indicators [20]. This comprehensive surveillance system aims to provide insights into population health dynamics within rapidly transitioning societies [20].

Encompassing an area of 420 km^2^, the HDSS spans 31 villages and encompasses 21,500 households, accommodating a population of 120,000 individuals [20]. The region is served by two health centres, seven government clinics, and three district hospitals, collectively striving to address the healthcare needs of the local community. The Bushbuckridge Sub-district confronts numerous challenges, including pervasive poverty exacerbated by high unemployment rates and a limited economic base, leading to significant labor migration. Acknowledging these challenges, the municipality was designated as a presidential nodal point in 2001 [21]. Many households rely heavily on government social grants as a primary source of income [21]. Moreover, the prevalence of HIV is substantial [2, 4], and there is a concerning upward trend in the incidence of non-communicable diseases among the population. The region is characterized by a significant presence of THPs, who play a prominent role in the local healthcare landscape [11]. Their influence and practices are deeply intertwined with the socio-cultural fabric of the community, offering alternative avenues for healthcare provision and healing [11, 22].

This study population comprised three primary groups originally recruited during a pilot study to assess the feasibility and acceptability of THP-led HIV counseling and testing. THPs were originally recruited to participate in the pilot trial if they were at least 18 years of age, lived within five miles of two study clinics, saw at least two clients per week, and were interested in partnering with the allopathic health system. We attempted to contact all THPs to complete interviews. Clients of THPs were recruited during the visit with their THP. If they agreed to participate, they were provided HIV counseling and testing, and if they tested positive, they were referred for allopathic care. We attempted to recruit all clients who received a positive test result to learn about their experience of receiving the test and linkage to care. Healthcare workers were purposively sampled based on their involvement in HIV-related work or treatment at clinics in the pilot study communities or their position overseeing clinical care across the sub-district. This group included stakeholders from non-governmental organizations (NGOs) and the Department of Health, including clinicians and nurses. All participants were 18 years of age or older.

## Procedures

The semi-structured, qualitative interviews took place in a private room or area at the health facility, at the home of the participant, or at a location of the participant’s choosing and took an average of 35 min to complete. Interview questions assessed: (i) opinions about, and/or experiences with, the THP-led counselling and testing intervention, including how this intervention fits into the cultural, social, and community values; (ii) community perceptions of clinic-based HIV counselling and testing, including cultural, social, or individual barriers to testing and treatment uptake; and (iii) perspectives on further integrating THPs into the health system, with a focus on cultural, clinic, community and interpersonal factors. The study team piloted the semi-structured interview guide to ensure that questions elicited an adequate response. The interviews were conducted by female and male trained interviewers (authors PM and WM), both with extensive experience in conducting qualitative interviews. They maintained a professional interaction with participants and used an emic approach to center on the participants’ points of view.

Interviews were conducted in the language most comfortable for the participant. THPs and their clients predominantly selected xiTsonga as their preferred language while healthcare workers predominately selected English as their preferred language. All interviews conducted in xiTsonga were transcribed and subsequently translated into English by the person who conducted the interview and double checked by one additional team member. All recordings were deleted. Interviews with healthcare workers continued until data saturation was achieved; those with clients and THPs were limited by those who participated in the original trial. Only two healthcare workers, who are facility managers at two different clinics, were unavailable for interviews during the designated time due to work and other commitments. Attempts were made to reschedule with those who were unavailable but were unsuccessful. We conducted interviews with only 11 of the 15 trained THPs as one had passed away and three could not be located. Only 10 of the 20 individuals who tested positive for HIV participated; one passed away, one declined, and eight could not be reached using the provided contact information.

### Data Analysis

The research team employed MAXQDA 2022, a qualitative data management software, to facilitate the analysis and synthesis of interview data. Two members of the research team (JH, PM) collaborated to develop a comprehensive code book. Subsequently, they conducted a phenomenological thematic analysis, prioritizing the participant’s perspective and how they interpret their experiences. The data were systematically categorized into overarching parent themes, including cultural norms, health facility/community behaviors, and relationships, using the socio-ecological model. This structured approach allowed for the rigorous examination and interpretation of the qualitative data, ensuring the extraction of meaningful insights and facilitating the identification of key patterns and recurring themes across participant responses.

## Results

### Demographic Data

We conducted 33 interviews, 12 with healthcare workers, 11 with THPs, and 10 with THP clients who received a positive HIV test result. Healthcare workers were 67% female, with a mean age of 46 years (SD: 8.56); THPs were 55% female, with a mean age of 46 years (SD: 8.21); and their clients were 70% female, with a mean age of 32 years (SD: 9.33). (Table 1)

**Table 1:** Demographic characteristics

	Female	Male
Traditional Health Practitioners (*n* = 11)		
Age (mean/SD)	(48.29/8.310	(39/6.07)
Education Level (Mean/SD)	(8.57/0.90)	(9.6/1.36)
Healer Patients (*n* = 10)		
Age (mean/SD)	(35/5.01)	(46.33/11.90)
Education Level (mean/SD)	(11/1.13)	(10/0.82)
Health Care Providers (*n* = 12)		
Age (mean/SD)	(48/6.78)	(43.25/6.94)
Type of Employment		
Nurse	3	0
Leadership/coordination position	2	4
Other	3	0

## Major Themes

The major themes discussed by participants included the importance of THPs within the context of South African society and how traditional beliefs often conflict with diagnoses and care associated with the allopathic health system. Lastly, community relationships, including those with THPs who live in rural areas, drive trust and culturally congruent care among those seeking HIV testing. THPs, if appropriately trained, are well positioned to reach community members who would otherwise avoid clinical services.

### Cultural Norms

Deeply rooted cultural beliefs were a significant factor driving people’s choice to seek care from a THP. Many individuals hold the conviction that certain ailments can only be effectively addressed by THPs due to their intimate understanding of cultural practices and spiritual beliefs. These cultural beliefs, consistent across healthcare workers, THPs, and their clients, served as a compelling motivator for individuals seeking care from THPs, highlighting the importance of respecting and integrating cultural perspectives within healthcare frameworks.

*Remember we are Africans and we cannot do away with that and the fact that we have different beliefs*,* values*,* and cultures so others do prefer to go to traditional healers and others do prefer to go to the health or modern system as the clinic*,* hence it is important for us to integrate the two so that they benefit both from the clinics and also from the traditional healers. And again*,* there are some conditions which they believe they can be treated traditionally and not in the health facilities. Hence it is important to integrate the two. (****NGO worker***,*** male****)*

These two types of conditions were further delineated by another healthcare worker.

*Tindzhaka* [traditional illness with similar symptoms to Tuberculosis but associated with violating cultural taboos], *they say it’s for traditional healers. And some also believe in cancer*,* they refer it to… what do they call it in Shangaan* [a specific tribe within the large Tsonda ethnic group, here the speaker is using it as a colloquial term for xiTsonga, the local language]*? There’s something that crawls in the skin and it penetrates deeper into the skin as it crawls…. Yes*,* they call it mfukuzani [*xiTsonga *word for cancer*,* often associated with bewitching]*,* because as cancer destroys whichever part of the body*,* they believe it’s mfukuzwani. So*,* things like cancer*,* and tindzhaka*,* people still believe that those are for traditional healers. “Swinhlokwana” in babies*,* they say babies have “nhloko”] the soft spot-on top of the head-fontanelle]. (****Department of Health clinical coordinator***,*** female****)*

*A healer confirmed this separation of traditional and allopathic conditions.*

*They [treatment with a THP and at the health facility] complement each other because…it is possible that a person that comes to me has TB and I will say it is “Mafularha” so this is why we work together with clinics. (THP*,* female)*

A THP client noted that the symptoms they experienced led them to believe they had an illness that needed to be treated by a THP. He noted that he *“was not feeling well in my body*,* always sleeping*,* and feeling tired. Felt as if I was bewitched.” (****HIV-positive client***, ***male****)* Thus, the perception of a difference between traditional and allopathic illnesses is not theoretical; people believe they can feel the difference and seek care accordingly.

### Health System

Two primary themes were prominent in our discussion with participants about the health system, HIV testing, and THPs. The first, focused on the challenges with the disrespectful behaviour of healthcare workers providing care, and their processes for conducting HIV counseling and testing. The second, focused on the challenges associated with integrating THPs into an allopathic system.

#### Staff Behaviour

Participants, both healthcare workers and people who had received an HIV test from a THP, recounted instances where they felt privacy was compromised at health care facilities, either due to breaches in confidentiality or disrespectful behaviour from healthcare workers.

*Yes*,* it’s experience from clinic. Where I come from there is this one nurse from [name of clinic] clinic whose primary role was HIV testing. Whenever she tests clients and as they come out*,* she would show a sign by hand which meant the person was HIV positive. This caused a lot of people to stop taking treatment from there and transfer to other clinics. (****HIV-positive client***, ***female****)*

*Okay*,* years back before I even went to nursing school*,* nurses were feared by many people and they wouldn’t even be open about why they were at the clinic because of how unfriendly the nurses were*,* our generation of nurses is very different. Then you’d go to the clinic because you have contracted an STI*,* but fear would make you say you’re at the clinic just because you have a headache. This is one reason why people don’t prefer coming to the clinics. Another reason would be that most people do not like to queue and there’s usually queuing at the clinics and people end up being seen by people whom they prefer not to be seen by. They also prefer getting their medication at Link Pharmacy or Clicks Pharmacy* [local pharmacies in the region], *some even go to see the doctors even when finance is an issue. This is to avoid queuing and the attitude that nurses give them. (****Professional nurse***,*** female****)*

Healers attempted to address this perceived concern of poor treatment at the health facility that was held by their clients.

*Our clients were usually very hesitant to test because they would say that they are not treated well in the clinics and that they talk about them. They give them their medications where everyone is looking (outside*,* not in a consultation room). So we were able to sit down and talk and I also went to the clinic to address the issue of people being afraid of going to the clinic to test because they say you give them their medications outside and I think this was resolved because when I went to collect my medication I saw changes so now people go to the clinics and I promised that I will address this issue with them at the clinic.****(THP***,*** female)***

Clients who had received a positive test from a THP, and had previously tested at a health facility, expressed dissatisfaction with the testing experience at allopathic healthcare facilities, highlighting challenges in how results were communicated. Many recounted instances where results were hastily explained, often in a rushed manner to accommodate the next patient, leaving them feeling confused and uncertain about their diagnosis. Inadequate explanations and rushed interactions may have contributed to misunderstandings regarding their test results, further exacerbating their distrust of the healthcare system.

*I went to two clinics where I was tested and the results from one clinic were positive*,* and the other were negative*,* so I asked her to test so I can be sure if positive or negative and the results were positive. I did not have a problem I just proceeded to initiate treatment. (****HIV-positive client***,*** female****)*

The second prominent theme associated with the allopathic health system was the process and challenges associated with integrating THPs into an already overstretched system. Resistance from allopathic providers emerged as a prominent challenge, reflecting concerns about the compatibility of traditional healing practices with established allopathic approaches.

*They (healthcare workers) need to stop with the attitude and understand that we (healthcare workers and traditional health practitioners) are colleagues. Everyone is doing their job. For instance*,* nurses in the clinic have different categories but we all see patients. I do not check vital signs because there is someone below me who does that. We shouldn’t be fighting about that but work hand in hand to get the job done and reach the goal. Nurses and doctors need to understand that the goal is to promote healthy lives. We shouldn’t undermine healers because they never went to school for 4 years. (****Professional nurse***,*** female****)*

*They used to not be very welcoming then*,* when our clients go to the clinic they would say stuff*,* whereas we are able to take our clients to them for checkup*,* but they do not allow the clients to come back to us*,* they would tell them to not come back to us. But it’s fine*,* now that we are working together*,* it is good. (****THP***,*** female****)*

The lack of institutional support posed as an obstacle to the formal recognition and inclusion of THPs within the healthcare system. Healthcare workers underscored the importance of providing proper training for THPs engaged in HIV testing to ensure the accuracy of results and adherence to safety protocols. Underlying these discussions was a combination of logistical concerns (e.g., distributing test kits and personal protective equipment) and the fear that THPs would not perform testing with fidelity, despite our study findings.

*They (traditional healers can collect at the clinic or the outreach team can reach out to them and help with the distribution……… and the clinics must also provide them with the containers and other utilities where they can properly dispose their equipment. (****Health care worker***,*** male****)*

*I think in terms of having them integrate into the system*,* it will assist us if we are going to train them under different guidelines that we are using and allow them to do practices before they can implement the program in the communities. They must also have a good relationship with the clinics so that should they encounter any challenges or problems*,* they call the clinic and get clarity. We have different modes or fields that we are referring to should we encounter any challenges. This will assist them (traditional health practitioners) to easily access information and when there are guideline changes or updates*,* they will get updated information through the clinic. (****Health care worker***,*** male****)*

All but one healthcare worker supported the involvement of trained THPs in HIV testing initiatives. She expressed skepticism regarding their capacity to conduct the testing effectively.

*Okay*,* isn’t it they don’t know [how to provide the test]? Even with the results*,* they don’t know how to read them. So*,* they will give the wrong results to the patient. They don’t even know where to keep the test kit for it to be safe for use so if the test is being placed in the wrong temperature or environment*,* it can give wrong results so it will…it’s a huge complication that one. (****Health care worker***,*** female****)*

While the study focused on HIV testing, healthcare workers expressed interest in moving beyond HIV testing and into the distribution of ART and TB testing.

*People are afraid to be judged by the clinic so it’s a good thing to bring services closer to people now that healers are testing for HIV. It’s a good thing. However*,* healers must also be able to give medication to clients who test positive*,* in that way*,* they won’t have to go to the clinic anymore. (****HIV-positive client***,*** female****)*

*… what I have realised is that a lot of our community members have TB and they do not consult until it is too late*,* and TB is fatal. Healers can offer health education about TB and that TB is curable*,* so people shouldn’t be afraid to go to clinics for treatment. Most people who go to initiation schools are usually infected with TB*,* some complain of swollen legs*,* and that is one of the signs of TB. This is why they get distended abdomen and eventually die from drinking the goat’s blood during initiation. HIV usually goes hand in hand with TB and the community may listen when healers educate them about TB compared to when we educate them because the healers are from the same community as them. (****Professional nurse***,*** female****)*

**Relationships with community members**,** including THPs.**

THPs are often perceived as more accessible than allopathic providers, particularly within their respective communities. Unlike formal healthcare institutions that may be distant or intimidating to some individuals, THPs operate within the fabric of the community itself. This proximity fosters a sense of trust and comfort, making it easier for individuals to seek HIV testing and counseling services from THPs. Their familiarity with local customs and traditions further enhances accessibility, ensuring that healthcare services are readily available to those who need them most.

*Yes*,* it does help a lot because clinics sometimes are far from where people stay but healers stay with us hence it should be easy to go to the traditional healer and testing can be done from there but if it’s something that requires further care at the clinic*,* the healer would be able to assist the client by hiring transport to the clinic. Some people’s situations are difficult so if traditional healers are testing that will make it easy. (****HIV-positive client***,*** female****)*

Additionally, THPs are often regarded as guardians of confidentiality, a crucial aspect of HIV testing and counseling. This perspective was shared by clients and allopathic providers. Unlike formal healthcare settings where privacy concerns may arise due to the presence of numerous staff members and administrative processes, THPs offer a more discreet and personalized approach to care. Clients feel reassured knowing that their personal information and health status will be kept confidential within the confines of the THP’s practice. This perceived assurance of confidentiality not only encourages individuals to seek HIV testing and counseling but also contributes to destigmatizing the process, ultimately facilitating greater uptake of essential healthcare services within the community.

*I think healers deal with confidentiality way better than the CHWs [community health workers]. I have never heard anyone complain that a healer has disclosed their clients’ issues to anyone before*,* be it abortion or just anything in general. I have never heard or come across such. (****Professional nurse***,*** female****)*

While THPs are well placed to support testing, they also have to navigate the difficult balance between providing spiritual/traditional care and moving into allopathic care. Largely, THPs have found this opportunity has increased their motivation and sense of accomplishment.

*It is good because even when I am sick or I accompany a client to the clinic*,* I am well-known there that I work from Wits*,* they even call me “Sister” when they see me*,* and also when I am wearing my Wits t-shirt*,* they come to me and ask me to pray for them in the morning in the waiting area*,* so this makes me really proud and I never expected any of this. (***THP**,** female***)*

While their self-worth has increased, they are still navigating expectations of their clients and the lack of protections.

*Yes*,* challenges were there. The challenge was that people are different and you need to be patient. … Some would understand and some would not*,* they would say that they did not come to me for that and we would lose clients but I would continue being patient… Initially it was hard because they like arguing and I wasn’t going to argue with someone that came to me for help because I could lose a lot or being spoken ill of*,* so I just took whatever and not say anything back. (***THP**,** female***)*

## Discussion

The findings of this study shed light on the intricate dynamics and deliberations surrounding the integration of THPs in rural South Africa into HIV testing and counseling programs. The themes that emerged from the analysis offer valuable insights into this complex intersection, with a focus on the potential for successful engagement and the impact THP participation would have on the health and wellbeing of the population.

In South Africa, traditional healing practices are deeply rooted in cultural beliefs and traditions, often serving as the first point of contact for individuals seeking healthcare [8, 9, 23]. Our study suggests that these cultural norms play a significant role in shaping community attitudes toward healthcare systems, including healthcare workers’ perceptions of THPs and their role in the delivery of clinical care in the region. Studies suggest that cultural competence and sensitivity are essential in effectively integrating THPs into formal healthcare systems [7, 12, 24]. Our study revealed that integration would also require some agreement on the types of services THPs could perform, the necessary training to achieve a skill level seen as acceptable to allopathic providers, and systems of oversight necessary to ensuring quality care delivery. Efforts to incorporate traditional healing methods into HIV prevention and treatment programs should be guided by a recognition of the cultural diversity and complexities within South African society [7, 9].

Studies across the continent suggest that THPs frequently encounter barriers, including stigma, discrimination, and a lack of recognition within the healthcare system [7, 24, 25], hindering their ability to collaborate effectively with allopathic providers. Healthcare workers in our study revealed similar concerns, despite a successful pilot study of HIV counseling and testing delivery. The lack of trust between THPs and nurses can pose a significant obstacle to patient care and effective collaboration [25]. Research indicates that initiatives focused on strengthening the capabilities of THPs, including HIV prevention and counseling training programs, play a crucial role in facilitating their integration into the healthcare system [9, 15, 18, 24]. In a Zambian study, community members emphasized the need for improvements in THPs’ qualifications, organizational practices, and adherence to good standards [26]. Our study highlighted a similar perspective. Progressing toward universal health coverage, the WHO recognizes the importance of traditional medicine and recommends integrating THPs into the healthcare system [17, 19].

In South Africa and other African countries, THPs are regarded as providers of culturally relevant care within a familiar environment [7, 13, 15, 18, 22, 27]. THP services are seen to respect cultural norms, beliefs, and language. Patients often feel more comfortable and validated seeking care from THPs who share their cultural background and worldview [11, 18, 22, 28, 29]. Our study revealed that clients were selective in terms of the individual from whom they wanted to receive their care, including HIV testing. The culture at the clinical sites did not allow this type of choice; patients were seen by the first available healthcare worker, potentially limiting clients’ willingness to seek care.

### Strengths and Limitations

Our study highlights the perspectives of THPs, their clients who received a positive HIV test result, and the healthcare workers who participated in our HIV testing pilot program in rural South Africa. By capturing data from each group, we were able to triangulate the successes, challenges, and opportunities such a program has to offer and how it fits into the cultural norms of South Africa. By working with individuals who had direct experience with our pilot study, we are not limited to speaking about a hypothetical partnership; people had a chance to experience THP-led testing and see the results before speaking their opinions. The study is limited by our inability to interview individuals who tested positive for HIV, or who delivered HIV testing but have either died, moved or are otherwise unreachable. We also opted not to interview those participants with a negative test result with the assumption that those with a positive test result would be better poised to describe their quality and value of the THP-led testing services. In addition, by enrolling only those who were part of, or familiar with, our pilot study, there may have been a response bias towards THP-led HIV counseling and testing. As a result, our study findings may not be generalizable to other regions where THPs have not historically been engaged in the system.

## Conclusions

Our study highlights the potential for integration and collaboration between THPs and healthcare workers in the context of HIV prevention and care. ​ By building trust, community members, THPs, and healthcare workers can work towards fostering more inclusive and collaborative approaches to HIV prevention and care. Providing THPs with appropriate training, materials, and support can lead to a more comprehensive and accessible healthcare system. ​.
